# Supplementary material for: Content-rich biological network constructed by mining PubMed abstracts
Source: BMC Bioinformatics. 2004 Oct 8;5:147. doi: 10.1186/1471-2105-5-147 (PMC528731; doi:10.1186/1471-2105-5-147)
Supplement: Additional File 5 — The original Chilibot query results of the term "long-term potentiation (LTP)" and 22 other terms, limiting the latest references analyzed to the years 1990, 1995, 2000, and 2004. [file 1471-2105-5-147-S5.bz2 › chilibotAdditionalFile5/ltp2004/html/TRKB_CAMKII.html]

 


 **TRKB** and **CAMKII** 
  
Found 4 abstracts in PubMed,  **4 abstracts were retrieved and analyzed**.  


---

 Search Google  |
 PDF files only 
|  EDU domain only 

---

**Interactive relationship** (e.g. stimulation, inhibition, etc)

- To examine functions of  **TrkB**  in the adult CNS,  **TrkB**  has been removed from neurons expressing  **CaMKII** , primarily pyramidal neurons, using Cre mediated recombination.  Ref: 10798407 Neuron, 2000

**Parallel relationship** (e.g. studied together, co-existance, homology, etc.)

- Data provide evidence that BDNF,  **TrkB** , and  **CaMKII**  alpha and beta mRNAs are accumulated in the dendrites of specific hippocampal neurons during pilocarpine seizures and kindling development.  Ref: 12121312 Epilepsia, 2002
- induction of  **CaMKII**  beta, BDNF, and  **TrkB**  mRNAs dendritic targeting in CA1, CA3, and DG neurons.  Ref: 12121312 Epilepsia, 2002
- induction of dendritic localization of  **CaMKII**  beta, BDNF, and  **TrkB**  mRNAs in CA3 neurons.  Ref: 12121312 Epilepsia, 2002
- Instead, the behavioral symptoms of  **trkB**   **CaMKII**  CRE mice, comprising hyper locomotion, stereotyped behaviors, and cognitive impairments, are similar to those postulated for mouse models of attention deficit disorder.  Ref: 14625139 Biol Psychiatry, 2003
- **trkB**   **CaMKII**  CRE mice cannot be regarded as a genetic mouse model of depression.  Ref: 14625139 Biol Psychiatry, 2003
- **trkB**   **CaMKII**  CRE mice showed a stereotyped hyper locomotion with reduced explorative activity, and impulsive reactions to novel stimuli.  Ref: 14625139 Biol Psychiatry, 2003
- METHODS Using the CRE loxP system, we bred  **trkB**   **CaMKII**  CRE mice with a  **trkB**  receptor disruption in the forebrain.  Ref: 14625139 Biol Psychiatry, 2003
- Nonstimulated rats BDNF,  **TrkB** , and  **CaMKII**  beta mRNAs localized in the soma and in the proximal dendrites of hippocampal pyramidal cells, and in the soma only of dentate gyrus DG granule cells.  Ref: 12121312 Epilepsia, 2002
- Fully kindled seizures no change in the subcellular distribution of BDNF,  **TrkB**  and  **CaMKII**  beta mRNAs.  Ref: 12121312 Epilepsia, 2002
- PURPOSE To analyze whether the subcellular localization of the messenger RNAs mRNAs coding for the neurotrophin brain derived neurotrophic factor BDNF, its receptor  **TrkB** , and the alpha and beta subunits of calcium calmodulin dependent kinase II  **CaMKII**  are modified after pilocarpine and kindled seizures.  Ref: 12121312 Epilepsia, 2002
